# Implementation of Health Promotion Competencies in Ireland and Italy—A Case Study

**DOI:** 10.3390/ijerph16244992

**Published:** 2019-12-08

**Authors:** Barbara Battel-Kirk, Margaret M. Barry

**Affiliations:** 1 BBK Consultancy, Omeath, A91RV10 Co Louth, Ireland; 2 World Health Organization Collaborating Centre for Health Promotion, National University of Ireland Galway, H91 TK331 Galway, Ireland; margaret.barry@nuigalway.ie

**Keywords:** Health Promotion competencies, evaluation, Health Promotion capacity

## Abstract

This paper reports on a case study that explored the broader contextual factors influencing the implementation of the CompHP Core Competencies at a country level in Ireland and Italy between 2011 and 2018. The sample comprised key informants who were Health Promotion experts and were knowledgeable about how the competencies had been used in their country. These experts formed National Reference Groups that guided the research process in each country and helped identify additional key informants. Qualitative methods were utilized consisting of a desk review and semi-structured interviews. The data from each country were analyzed separately using a thematic analysis approach, with the findings then compared and reviewed by the National Reference Groups. A total of 26 interviews were completed (13 in each country). The findings show that both the focus and rate of progress of implementing the competencies differed across the two countries and that this reflected their levels of Health Promotion infrastructure and capacity development. A lack of awareness of the competencies was identified as a major limiting factor in implementation in both countries, of particular concern in relation to employers and decision-makers. While the case study focused on implementing the competencies in two European countries, there are insights from their experience that can inform implementation in other countries. The study also begins to address the gap in empirical evidence on the use and impact of Health Promotion competencies and the factors that influence their implementation.

## 1. Introduction

Identifying and agreeing core competencies has been acknowledged as an essential component of developing and strengthening Health Promotion workforce capacity to improve health and wellbeing [1,2,3]. It was in this context that the CompHP Core Competencies Framework for Health Promotion [4,5] was developed in 2012 as the core element of a European Union funded project that developed competency-based quality assurance for Health Promotion practice, education and training in Europe [6].

As a rationale for developing competencies, reference is made in the international literature to the many positive benefits that can accrue from their implementation. These include enhancing Health Promotion workforce capacity, quality assurance of practice, education and training, and as a basis for developing a shared vision of what constitutes the specific knowledge and skills required for effective and ethical Health Promotion practice [1,2,3,4,5,6,7,8]. However, potentially negative consequences of competencies have also been identified, including the fact that they may undervalue professional judgement and restrict the dynamic nature of Health Promotion practice [9,10,11].

Despite these divergent opinions on Health Promotion competencies, there is a paucity of empirical evidence available on the actual use of competencies or their impact and whether the beneficial or negative outcomes attributed to them can be validated [7]. It is in this context that an evaluation of the use and impact of the CompHP Core Competencies on Health Promotion practice, education and training in Europe was initiated in 2016.

Stage one of the evaluation comprised a scoping review of the literature and research on Health Promotion competencies [7], followed by an online survey that explored attitudes towards, and the use and impact of, the CompHP Competencies across the European Health Promotion community [12]. This paper reports on the final stage of the evaluation process comprising a case study focusing on the implementation of the CompHP Competencies at a country level in two European countries, namely Ireland and Italy.

### Background

While there are references in the literature to the development of Health Promotion competencies since the mid-1980s only two examples of their empirical evaluation were identified in a recent scoping review [7], both of which were undertaken in New Zealand [13,14]. The findings from these small studies [13,14] indicated that the competencies had been used in different contexts and settings and that reactions to their implementation were positive.

A number of factors that are considered likely to influence the implementation of the CompHP Competencies have been identified [7,8,15], including levels of Health Promotion infrastructure and capacity, attitudes to their usefulness and relevance, recognition of Health Promotion as an area of professional competence, availability of support and resources and political, organizational, professional and educational contexts.

In an online survey of 81 Health Promotion specialist from 25 European countries, Battel-Kirk and Barry [12] found that respondents’ attitudes to the competencies were mainly positive. However, only 53% of respondents reported that the competencies were actually used in their country and 54% indicated that they used them in their practice. The survey findings provided some insight into the factors that influenced individual decision-making in using the competencies but also identified a lack of recognition and support by key organizations and stakeholders at a country level as potentially critical factors in their implementation.

It was in this context that the third stage of the evaluation was initiated comprising a case study of factors influencing country level implementation. The overall aim of the study was to explore and compare the broader contextual factors influencing the implementation of the CompHP Competencies at country level in Ireland and Italy between 2011 and 2018. The case study had the following specific objectives: (i) to describe the contexts within which Health Promotion is implemented in each country; (ii) to explore perceptions of readiness to implement the competencies at country level; (iii) to review the implementation and impact of the competencies; (iv) to investigate the factors that influence the implementation of the competencies in both countries; (v) to compare the findings from both countries in the context of future implementation of the competencies at national and international level.

## 2. Materials and Methods

A single case study was employed with two embedded units of analysis, namely the countries of Ireland and Italy. A case study method was chosen as it is an approach that is ideal for investigations that aim to move beyond narrow definitions of a research topic, address the context rather than isolated variables, and incorporate multiple sources of evidence [16,17,18]—all of which apply to this study. The study encompassed both ‘theory-testing’ and ‘theory-building’ aspects [17] as it explored theories surrounding the implementation of the competencies identified in the literature while being open to the emergence of alternate theories.

The study was bounded by geography and time, (i.e., the implementation of the CompHP Competencies in Ireland and Italy between 2011 and 2018), with the rationale for concentrating on these countries being that

- there was evidence of implementation of the competencies in both countries [15,19].
- the highest number of responses to the online survey were received from respondents in these countries [12]
- there were known differences between the countries in terms of Health Promotion infrastructure and capacity [7,15], allowing for a useful comparison.

The design of the case study was informed by consensus standards for the reporting of case studies that aim to improve the consistency, rigor and reporting of such research [20]. The case study also drew on the Consolidated Framework for Implementation Research (CFIR) [21], as a conceptual framework to guide the assessment of the multilevel implementation of the CompHP Competencies in both countries and to identify the main factors that influenced the level of implementation achieved [22,23,24,25]. The CFIR is intended to be tailored to the specific context being studied, with Alexis Kirk et al. [25] recommending that the constructs selected and the rationale for their selection be clearly stated.

The CFIR constructs selected for this study were those considered to ‘best match’ its aim of exploring implementation of the CompHP Competencies at country level, i.e.,

- the ‘outer setting’ construct with particular reference to Health Promotion infrastructure and capacity at country level.
- the ‘inner setting’ construct applied at country level with emphasis on the subconstructs of readiness to implement resources and support.
- the implementation process construct, including the subconstructs of planning, use, champions, leadership, reflection and evaluation.

Based on Yin [18], all aspects of the study were triangulated by

- accessing evidence from different sources (i.e., documents, reports, informants, national experts).
- using different methods (i.e., documentary analysis, interviews and thematic analysis).
- including national experts’ comments in the final analysis of findings.

Collection and analysis of the data from each country were conducted separately, and the findings were then compared.

### 2.1. Sample

The sample for the case study initially comprised five known experts in each country who were knowledgeable about the contexts within which Health Promotion is practiced and how the CompHP Competencies had been used in their country. These experts were invited to act as key informants and served as national reference points for the study. The experts included leading members of national Health Promotion professional and academic bodies and others in Health Promotion/Public Health departments and statutory/non-governmental organizations with a remit for Health Promotion at country level (and at regional level in Italy). The experts assisted in piloting interviews, participated in interviews, commented on findings and assisted in identifying additional key informants to be interviewed in their country, with the aim of focusing on those most likely to share relevant information rather than identifying a specific number of informants [18].

### 2.2. Methods

The case study employed qualitative methods comprising a desk review and semi-structured interviews with key informants.

### 2.3. Desk Review

A desk review was undertaken to collate data on the Health Promotion policy and practice contexts within which the CompHP Competencies were implemented in both countries. Sources included key policy documents, reports and articles published since 2005 that were available in full, or in summary, in English. Sources were identified through a rapid review of the literature, internet searches and recommendations from the National Reference Groups.

### 2.4. Semi-Structured Interviews

Semi-structured interviews were the main data collection method employed. The interview protocol was developed using selected constructs from the CFIR Interview Guide Tool [26] together with key issues from the from the existing literature [7,15], the online survey [12] and the desk review. Those agreeing to be interviewed were assured of confidentiality and anonymity and were asked to sign a form indicating that they consented to their interview being recorded.

The interview questions and process were piloted with two informants from each of the two National Reference Groups in January 2019, resulting in some minor revisions. The remaining interviews were conducted between January and March 2019 using online meeting tools in most instances. Interviews were recorded with the informants’ permission and conducted in English. An outline of the interview questions was emailed to informants 10 working days before their interview.

### 2.5. Collation and Analysis

Interviews were transcribed by the main researcher and following the thematic analysis approach described by Braun and Clarke [27], the data from each country were read through a number of times to allow for familiarization. Initial codes were then generated and emerging themes identified, refined and named in consultation with the second researcher.

Analysis focused on identifying themes emerging from the data from each country separately, with the emergent themes then collated and interpreted with reference to the selected CFIR constructs, factors identified in the literature and findings from the desk review. The weighting attributed to the themes was informed by how frequently that informants referred to each theme and the emphasis they placed upon specific issues in the interviews.

The initial findings from each country were then compared and reviewed by the National Reference Groups, with their expert feedback informing the results presented below.

## 3. Results

### 3.1. Desk Review

A summary overview of the findings from the desk review concerning the Health Promotion context in each country is first provided.

Ireland and Italy are both parliamentary democracies and members of the European Union. The countries vary greatly in size and population, i.e.,

- *Land area*—Ireland 69,800 km^2^; Italy 302,100 km^2^
- *Population*—Ireland 4.63 million; Italy 60.8 million [28]

Ireland has a centralized health system commonly referred to as ‘two-tiered’ as many individuals buy private insurance to bypass long waiting lists in the public system and gain faster access to diagnostics and hospital treatments. Ireland remains the only western European country without universal coverage for primary care [29]. In 2019 approximately 46% of the population had some form of private health insurance plan [30].

Health policy is determined by the Department of Health [31] and health service delivery is managed by the Health Service Executive (HSE) [32].

Italy’s health care system operates both at national and regional level, as since the early 1990s, regional governments have devolved autonomy in planning and organizing healthcare in their territory, with the level of autonomy described as very high [33]. Universal coverage is largely free of charge at the point of delivery but approximately 30% of the population has additional private health insurance [34].

Findings on Health Promotion infrastructure and capacity in Ireland and Italy are presented in Table 1.

### 3.2. Interviews and Informants

A total of 26 informants were interviewed, comprising 13 national experts in each country, including members of the National Reference Groups. As the Health Promotion community in both countries is relatively small, the number of experts interviewed from specific settings and roles is not specified in order to maintain anonymity. Those interviewed included Health Promotion academics, practitioners, managers and representatives of professional associations in both countries. In Italy, informants were from national and regional levels, with the majority from the academic and training sectors. Informants in Ireland included those employed at policy and practice level and in academic, statutory, nongovernmental organizations (NGOs) and the private sector.

Three Italian informants responded in writing as they considered that their English conversation skills were not adequate for their participation in an interview.

### 3.3. Implementing the CompHP Competencies in Ireland and Italy

The findings are reported separately for each country and with reference to the CFIR constructs/subconstructs selected, i.e., readiness to implement, use and impact of the competencies, and key factors influencing their implementation.

#### 3.3.1. Readiness to Implement—Ireland

Differing levels of readiness to implement the competencies were reported across different settings, with most informants indicating that academic organizations were at maintenance stage, the professional association/NAO was at action stage but that readiness at practice level was still at an early level. Overall, most informants considered that there had been good progress nationally:

‘on a scale of 1-10 Ireland is at 6 … people realize that this is what we need to define our role.’

‘at least 50/50 willingness/readiness to implement the CompHP Competencies nationally.’

However, a few informants considered that readiness was

‘maybe contemplation stage … parts of Ireland where they are just thinking about it.’

#### 3.3.2. Readiness to implement—Italy

A large majority of Italian informants reported that readiness to implement the competencies was at very early stages across all settings:

‘very early in process … slow and difficult to involve people at national and regional level … a lot of work’

‘we need wider diffusion about them and more time to do that before being ready to implement.’

#### 3.3.3. Implementation—Ireland

Formal implementation of the competencies was reported in two main contexts, i.e., in the introduction of a of professional registration system and associated Continuing Professional Development (CPD) activities managed by the Professional Association [45], and in the context of university degree programmes that have been accredited under the IUHPE Accreditation System for Health Promotion education and training. The competencies were also reported as having strongly influenced the development of a number of new Health Promotion courses at both undergraduate and postgraduate levels.

While there was little evidence of formal implementation of the competencies at an organizational level elsewhere, some informants indicated that they used the competencies in their individual practice, often on an ‘ad hoc’ and ‘implicit’ basis (i.e., without formal reference to the CompHP Framework).

#### 3.3.4. Implementation—Italy

Formal implementation of the competencies was reported in the context of two university degree programmes that have been accredited by the IUHPE.

Reference was also made to their use in Health Promotion education in other professional academic courses (e.g., medicine, psychology) and in training for health professionals at regional and national level. In addition, the competencies were also used for informing the development of an online ‘best practice’ framework [52] and regional research on Health Promotion workforce development.

Details of how the competencies were used, other than in accredited courses, included that they were sometimes ‘mixed and matched’ with other frameworks, adapted to local contexts and that use was often ‘implicit’:

‘I do not name the CompHP framework … I talk about them, but I do not name them.’

#### 3.3.5. Impact

While no formal evaluation of the impact of the competencies had been undertaken in either country, all informants stated that, on reflection, their experience of implementing them was very positive. Most informants reported impact on the quality of education and training, recognition of Health Promotion and improved quality assurance (see Table 2).

### 3.4. Factors Influencing the Implementation of the CompHP Competencies in Ireland

**Figure 1:**
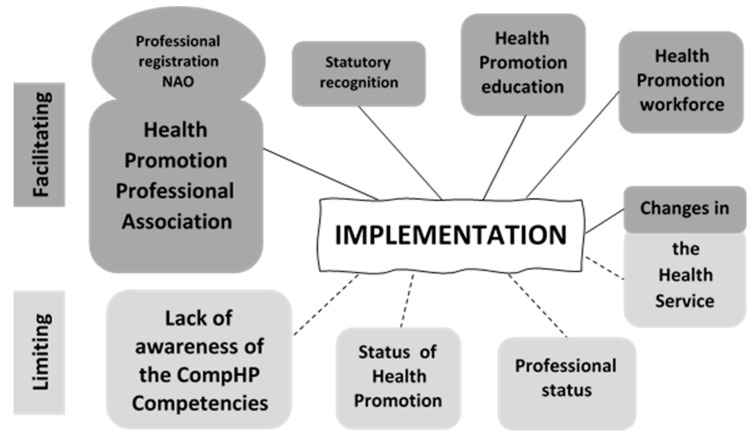
Main factors facilitating and limiting implementation of the CompHP Competencies at country level in Ireland.

The main themes emerging from the data from Ireland in relation to factors influencing the implementation of the CompHP Competencies are illustrated in Figure 1. The size of the shapes in the figure reflects the weighting attributed to the themes based on how frequently informants referred to each theme and the emphasis they placed upon specific issues in the interviews.

### 3.5. Factors Faciliting Implemention in Ireland

#### 3.5.1. Health Promotion Professional Association/National Accreditation Organization (NAO)

Informants across all sectors acknowledged the very significant role that the Health Promotion Professional Association had played and continues to play in implementing the competencies, for example:

‘the Association has done hugely valuable work on the CompHP Competencies … there is a momentum behind it.’

The role of the Association in establishing a NAO, with a remit to register Health Promotion on the practitioners in Ireland on the IUHPE Accreditation System was viewed as a major boost in implementing the competencies:

‘as you see more registration you will see the CompHP Competencies becoming more embedded in people’s thinking and practice.’

In developing the NAO, the planning process was described as ‘*slow and inclusive’* and its success was attributed to a ‘*really good team’* involving ‘*keen and motivated people*’. Informants made reference to the support provided by the IUHPE, champions within the Health Promotion community and small amounts of funding from statutory bodies as contributing to the successful development of the NAO.

However, a lack of resources was highlighted as a potential limiting factor going forward, as the Professional Association and NAO operate on a voluntary basis.

#### 3.5.2. Potential for Statutory Recognition

Implementing the competencies and establishing voluntary professional registration were viewed as a positive step towards gaining statutory recognition for Health Promotion:

‘to have our own competencies and registration in place is a good stepping-stone towards statutory with CORU (https://www.coru.ie/) and registration down the line.’

Participants expressed the view that as registration grew, there would be increasing momentum towards establishing statutory recognition, although this was unlikely to happen in the near future.

#### 3.5.3. Health Promotion Education

Most informants made reference to the positive impact of the implementation of the competencies through Health Promotion education, with a majority indicating that they had become aware of them as students in Health Promotion courses and/or through an annual Health Promotion conference hosted by an academic institution. It was considered that

‘the more embedded and centered work and experiences is around the CompHP Competencies at the educational level the more widely understood and used they will become.’

The leadership role played by Irish academics in the development of the competencies and their ongoing support were regarded as important factors in their implementation.

Graduates of accredited courses were also recognized as potential champions for the competencies.

#### 3.5.4. Health Promotion Workforce

Frequent reference was made to the usefulness of the competencies in the context of a specialized workforce, e.g., in gaining recognition for and defining roles, assuring quality and underpinning education and informing CPD:

‘I think a competency framework is like a safety blanket or a protection and definition of what we do in a very succinct way.’

Despite concerns about the level of awareness of the competencies, there was evidence of support for their implementation in the workforce:

‘people who are working on the ground are keen for this (implementation) to happen … they see the value in the CompHP Competencies.’

### 3.6. Factors both facilitating and limiting implemention in Ireland 

Organizational Changes in the Health Service Executive (HSE)

Organizational changes within the health service were identified as having both facilitating and limiting aspects in terms of the implementation of the competencies. Informants across all sectors stressed that the position of Health Promotion within the HSE, the largest employers of Health Promotion practitioners in Ireland, was a pivotal factor in implementing the competencies nationally.

However, what was described as ‘constant organizational change’ within the health service in recent years, ongoing as interviews were conducted, was considered by some as potentially threatening the sustainability of Health Promotion roles and functions. Some informants suggested that the competencies served as an authoritative source when arguing to maintain capacity:

‘I think the competencies can offer a context for a conversation that might need to be had in terms of what the role and function of Health Promotion is in the new structure.’

Changes in leadership in the organization were considered to offer opportunities, but also pose challenges:

‘the national leadership in Health Promotion in the service will have a much different role … so maybe there is an opportunity for more buy-in of the competencies … equally there might be more resistance.’

While some informants believed that the competencies were not recognized by senior level health service managers, the fact that they had recently been referenced in the recruitment and selection of new Health Promotion managers was viewed as a positive step in this regard.

### 3.7. Factors Limiting Implmentation in Ireland 

#### 3.7.1. Lack of Awareness of the CompHP Competencies

Most informants reported that limited awareness of the competencies across all levels and settings was a major barrier to their implementation, in particular in relation to employers and decision-makers.

Practitioners who were not involved with the Professional Association and/or who work outside the capital city were identified as being less likely to be aware of the competencies.

Some considered that there had been more awareness of the competencies when they were first developed, and it was strongly emphasised that a more targeted and sustained dissemination of information was required.

#### 3.7.2. Status of Health Promotion

The status of Health Promotion at policy level was highlighted as a pivotal factor in implementing the competencies:

‘where Health Promotion is ‘at’, at a policy level, makes a big difference in implementing them as all funding comes through the policy stream.’

Some informants referred to a ‘*shift*’ from the ‘*settings approach’* to a ‘*health and wellbeing’* approach in the current ‘Healthy Ireland’ policy [35], with Health Promotion being integrated into the wider health and social care agenda. This shift was linked to changes in terminology fostering fears that, if Health Promotion was not specifically referenced in funding streams and national policies, there could be negative repercussions for the relevance of the competencies:

‘The term Health Promotion is not used anymore in the health service … it’s now ‘health and wellbeing’… this hinders the uptake of the competencies and registration.’

‘In our organization (NGO) we have a prevention department … it used to be called the Health Promotion department … not sure what that means for the competencies.’

Limitations on funding for Health Promotion at national level, associated with political imperatives to focus on acute care services were also considered as likely to have a negative impact on implementing the competencies.

However, a few informants considered that the situation for Health Promotion had improved with ‘*more reference to Health Promotion in government strategies and more jobs’*, resulting in a more positive environment for implementing the competencies.

#### 3.7.3. Professional Status

The fact that Health Promotion is not formally recognized as a profession in Ireland was believed by many to have negative implications for the competencies, as

‘it appears that anybody without a professional qualification in Health Promotion or without experience is entitled to, and has the ability to, do Health Promotion.’

Professional status was regarded as being of particular relevance for Health Promotion practitioners in the health service as their employment is currently graded as administrative rather than professional:

‘the competencies would need to be linked to grading for our profession and that can be seen as been helpful (to implementation) but also as a hindrance as it hasn’t happened.’

For all informants, implementing the competencies was viewed as useful when making the case for professional status.

### 3.8. Factors Influencing the Implementation of the CompHP Competencies in Italy

**Figure 2:**
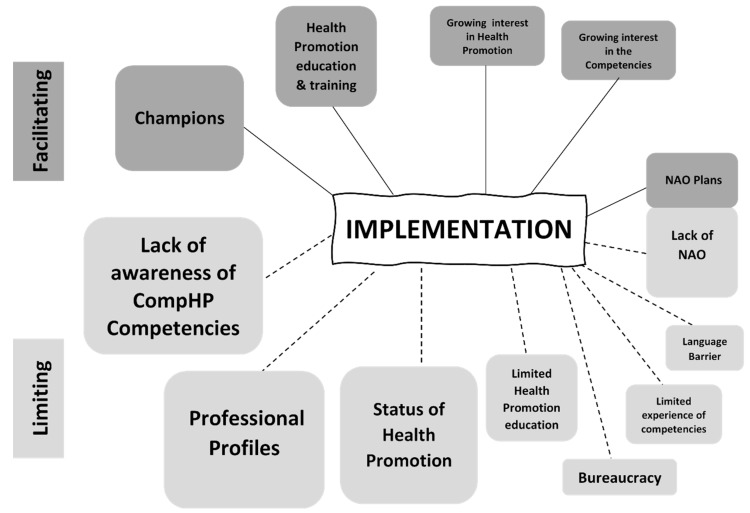
Key factors facilitating and limiting implementation of the CompHP Competencies at country level in Italy.

The factors influencing the implementation of the CompHP Competencies emerging from the Italian data are illustrated in Figure 2**.** The size of shapes in the figure reflects the weighting attributed to the themes based on how frequently informants referred to each theme and the emphasis they placed upon specific issues in the interviews.

### 3.9. Factors Faciliating Implementation in Italy 

#### 3.9.1. Champions

Many references were made to key people in the Health Promotion community in Italy who had shown leadership and championed the development and implementation of the competencies. Some informants also highlighted that they, and their organization, were championing the competencies:

‘when we speak about Health Promotion on every occasion we use the CompHP Competencies.’

Graduates of accredited Health Promotion courses were also regarded as future champions for the competencies.

#### 3.9.2. Health Promotion Education and Training

The importance of Health Promotion education and training was also identified and it was suggested that

‘the demand for the competencies will progressively increase as a consequence of more effective Health Promotion training and universities courses.’

Those currently implementing the competencies in education and training were regarded as role models for others wishing to develop competency-based curricula. Where the competencies had been implemented in educational settings it was reported that managers had been supportive and that people were the main resource used.

Education and training organizations’ role in disseminating information on the competencies was also acknowledged, e.g., through newsletters (e.g., [51,52]), peer-reviewed articles [53] and knowledge transfer via a ‘best practice’ website [52].

#### 3.9.3. Growing Awareness of Health Promotion and the CompHP Competencies

While the predominance of prevention and the ‘medical model’ of public health was stressed, there was reference to a growing interest in Health Promotion approaches in some regions and national organizations, with the competencies having a potential role in supporting this:

‘recognition of Health Promotion will reach new levels if the CompHP Competencies work to change, or at least begin to change, how the regions operate.’

The recent decision of the Ministry of University and Research to include Health Promotion as a topic within postgraduate courses for non-medical health professionals was also offered as an indication of increasing awareness of, and support for, Health Promotion at national level.

Similarly, despite concerns about a general lack of awareness, some informants pointed to evidence of growing awareness of, and interest in, the competencies:

‘the people we work with … like regions, groups, colleagues … they are very, very interested in the competencies.’

### 3.10. Factors both Facilitating and Limiting Implementation in Italy 

#### Plans for Developing/Lack of a National Accreditation Organization (NAO)

The issue of developing a national professional body or NAO was viewed as having both facilitating and limiting influences on the implementation of the competencies. For example, it was indicated that if ongoing plans to establish a NAO came to fruition, this would give impetus to implementing the competencies.

However, while formal plans had been drawn up, some funding secured and some progress made in establishing the partnership that will form its governing body, the ongoing lack of a NAO was identified by over half of informants as an obstacle to implementing the competencies. Progress on the development of the NAO was described as ‘*difficult and slow*’ with ‘*difficulties in engaging people’*.

Resistance to the development of a NAO was also reported:

‘there is a lot of resistance to that … because to people who work in in public health Health Promotion is something that is regarded as a natural part of the public health profession … not a competency that you have to support and improve.’

Concerns were also expressed about the potential for inequities in relation to the NAO:

‘the richer regions will get the System … you will see many regions from the north and less from the south involved.’

Other difficulties identified in setting up a NAO included a lack of support from decision- makers, overall bureaucracy, and limited resources. The experiences of those who had developed a NAO in other countries and the findings from this case study were suggested as ways to inform the development process.

### 3.11. Factors Limiting Implementation in Italy 

#### 3.11.1. Lack of Awareness of the CompHP Competencies

All informants were of the opinion that there was very little awareness of the competencies across all levels in Italy and that this was a major block to their implementation:

‘So far the competencies are not much known and haven’t really influenced at national level’

‘Some aware … but a few people … there isn’t a critical mass for change at national, regional or local level or within the university.’

It was reported that very few employers were aware of the competencies, although they are a key group that need to be involved in their implementation and it was emphasized that is a situation that needs to be addressed.

Awareness of the competencies overall was summarized as

‘they are not widely known … little spots in different places and these are not linked together.’

#### 3.11.2. Professional Profiles

The fact that there is no specific Health Promotion professional profile, combined with the strict statutory regulation of professions and meticulous delineation of professional boundaries in Italy, were identified as major limiting factors in implementing the competencies in practice, education and training:

‘it’s the legal title of the degree … so you can do a profession only if you have this degree … it’s very difficult … it would be an enormous change in legislation (to recognize Health Promotion) … I don’t think it is possible’

‘we cannot ask other people to embrace them (the competencies) because they have other profiles.’

As most recruitment calls for employment in the context of Health Promotion require that the applicant be registered in a statutorily recognized profession, it was argued that

‘you can have Health Promotion registration and know the competencies but unless you have registration and education in a defined area you are not able to apply for public calls.’

Doubts were also expressed about the possibility, or even the desirability, of statutory recognition of Health Promotion:

‘I don’t think it’s possible to create a Health Promotion practitioner register ... as a profession … because we have a particular legislation about the different professions … most of the people are not agreeing with this kind of definition … to recognize Health Promotion … because different professionals are involved in Health promotion and each single profession has a special register so it’s very, very difficult.’

This situation was viewed as part of a wider problem as

‘some employers have difficulty recognizing nursing or physiotherapy as a profession like medicine … we are still very medically oriented … so it’s very complicated to work on Health Promotion … but it’s not only with Health Promotion.’

There was also little support evident for a Health Promotion specific workforce:

‘Health Promotion professionals … it’s not so widespread in Italy … I think it is better to have a specific Health Promotion module for psychologists, for medical or education practitioners.’

It was suggested that in the Italian professional context, the best way forward might be to emphasize the value of the competencies as a quality assurance framework and competency-based registration as a valuable ‘*added*’ title for practitioners with other professional titles.

#### 3.11.3. Status of Health Promotion

Many informants reported that Health Promotion was not well understood, supported or implemented at national and regional levels, and that this created barriers to implementing the competencies:

‘knowledge is very low about Health Promotion and lots of people confuse Health Promotion with prevention or Health Education … so it’s very, very difficult about the competencies.’

Some informants considered that the national policy was overly focused on prevention while others argued that it was the application of the policy across a devolved health system that was problematic, with reports of a stronger commitment to Health Promotion in northern regions.

Many informants reported that prevention and health education actions were often mis-labelled as Health Promotion, with suggestions that this should be challenged referencing the competencies as to what constitutes Health Promotion.

#### 3.11.4. Language Barrier

Although the competencies have been translated into Italian [54], a number of informants identified a language barrier in implementing them and viewed developing a NAO as a way to overcome this:

‘if they (the competencies) remain in English and the registration is in English … we have a few people only in Italy (able) to use them.’

‘we will have a NAO for accreditation in Italy because the language is a big wall.’

It was also suggested that the current translation of the competencies should be reviewed with particular attention paid to translating key concepts:

‘we have difficulties even in translating the word competencies … we should start with what is there and go through it slowly and improve on it.’

#### 3.11.5. Limited Health Promotion education

A lack of Health Promotion education and training was viewed as a factor limiting the implementation of the competencies, but they were also suggested as an authoritative source when arguing for more, and better quality, education and training.

#### 3.11.6. Bureaucracy

Bureaucracy was identified by a number of informants as a reason for slow progress in implementing the competencies:

‘In Italy it’s a slow process related to the characteristics of the system … not only the competencies … the system overall is very slow … bureaucracy is a problem.’

#### 3.11.7. Limited Experience of Competency-Based Approaches

A few informants suggested that the fact that there was not much experience of using competency-based approaches in general may create a barrier when implementing the competencies, but one informant reported:

‘Competencies as a professional discourse is coming into the professional culture’.

### 3.12. Future Implementation of the CompHP Competencies

Informants responses regarding what advice they would give to others who intended to implement the competencies are summarized in Table 3.

Finally, an Italian informant suggested that

‘The competencies need to become the mentality of the person so that when they use them, they talk to people with passion.’

## 4. Discussion

The findings from the case study show that while the CompHP Competencies have been implemented in different settings and contexts in both Ireland and Italy, the modes and rates of progress of implementation differ. The findings also suggest that the levels of Health Promotion infrastructure and capacity at a country level are important influencers in the implementation of the competencies.

Many of the factors identified as influencing the implementation of the competencies in both countries are similar to those that have been previously identified in the literature, for example, in terms of infrastructure (Health Promotion status/policy) and capacity (workforce, professional profiles/recognition, education and professional associations).

The influence of differences in levels of Health Promotion infrastructure and capacity was evident in the themes emerging from the data across the CFIR constructs and subconstructs selected to inform the study. For example, there is evidence of readiness to, and good progress in, implementing the competencies in Ireland, where there is a history of Health Promotion specific policies and well established Health Promotion capacity, including a specialized workforce, a dedicated national Professional Association and specific education programmes since the 1990s. In contrast, in Italy, where policy is more focused on prevention, Health Promotion is seen as being part of the role of other professionals rather than a specialist Health Promotion workforce and Health Promotion education is limited, readiness to implement the competencies was reported as being at a very early stage and progress as slow and difficult.

The use of the competencies in both countries was also found to reflect their levels of Health Promotion infrastructure and capacity. For example, while formal implementation of the competencies in the context of accredited Health Promotion courses in both countries was similar (three in Ireland, two in Italy) this finding should be viewed in the context of a much larger academic sector in Italy, reflecting the much greater population.

There were differences between the countries in the formal implementation of the competencies in the context of national professional bodies and NAOs within the IUHPE Health Promotion Accreditation System. In Ireland, a major focus of implementation has been the development of a NAO, with the Health Promotion specific Professional Association taking the lead on its development and a specialized workforce showing interest in becoming registered practitioners. In Italy, the slow process in establishing a NAO was linked to difficulties in developing a partnership of stakeholders as a governing body and the fact that registration was viewed as an ‘added extra’ for health professionals rather than as a system of professional recognition for specialized Health Promotion practitioners. In addition, there were indications of opposition from some in public health in Italy as Health Promotion is not recognized as a distinct profession with its own competencies, a situation that was not evident in the Irish data.

While there were some commonalities across the findings from the two countries on the factors considered to influence implementation of the competencies, more positive factors were identified in Ireland, as might be expected given its more advanced levels of Health Promotion infrastructure and capacity.

For example, in Ireland the Health Promotion specific Professional Association was identified as a main facilitating factor, reflecting established Health Promotion capacity that could provide leadership and organizational support in implementing the competencies at a country level. In contrast, in Italy, the main facilitating factor identified was individual champions, likely indicating the need to develop and strengthen Health Promotion capacity and structures within which to formally implement the competencies.

There were also commonalities in the limiting factors identified in both countries, in particular, a lack of awareness of the competencies at all levels. While some useful examples of dissemination of information on the competencies were identified, there was reference in both countries to limited resources for dissemination, together with a lack of consensus on how to inform key groups, notably employers.

While the status of Health Promotion was viewed as a limiting factor in both countries, the underlying reasons were different. In Ireland, the focus was on a change in approach in a well-established Health Promotion policy context, while in Italy, concerns were focused on a national policy more centered on prevention and a lack of knowledge and understanding of Health Promotion. However, there was recognition in both countries that the competencies have a role to play in both developing and maintaining the status of Health Promotion. It was interesting to note that ongoing organizational change in the health service in Ireland and resistance to change in Italy featured as factors limiting the implementation of the competencies in each country. It is likely that the rate and process of implementing the competencies going forward will reflect not only the level of Health Promotion infrastructure and capacity but also the organizational culture within which they are implemented.

With regard to the implications of the findings for future implementation, it is clear from the data that a lack of awareness of the competencies was viewed as a major limiting factor by informants in both countries, in particular with reference to employers and decision-makers. This overall lack of awareness, in addition to a lack of support for, and formal recognition of, the competencies by key organizations and stakeholders are major issues for future implementation that and will need to be addressed. Irrespective of any other influencing factors, lack of awareness could potentially undermine future implementation, not only in Ireland and Italy, but globally. This finding highlights the need for more targeted and sustained information-sharing and marketing of the competencies at all levels and greater advocacy to ensure their implementation.

Another factor with implications for future implementation is the professional status of Health Promotion. While Health Promotion is not a statutorily regulated profession in either country, the issue of professional regulation impacted on implementing the competencies to a much greater extent in Italy and this has implications for the focus on implementation going forward. As acknowledged when the competencies were being developed, while their key target audience is Health Promotion practitioners, they could be useful to other health professionals in countries where there was no specialized Health Promotion workforce, and this has proved to be the case in Italy. Similarly, the recommendation that the competencies could be used as ‘stand-alone’ quality standards in countries with less well-developed Health Promotion infrastructures was suggested as a possible way forward in Italy.

The issue of language as a barrier to implementing the competencies was raised in Italy, with added emphasis on the fact that translation is challenging given different cultural and linguistic interpretations of key concepts and core words in the competency context. These difficulties reinforce the need for translation of the competencies to be a cooperative endeavor between skilled translators and experts with a solid grounding in Health Promotion and also for the need for future research to be conducted in informants’ first language.

The findings from Ireland and Italy demonstrate that an understanding of the policy, practice and organizational contexts within which the competencies are implemented will help identify how best to proceed. Whatever the context, raising awareness of the competencies at all levels is a key first step, an exercise that should be tailored to the specific cultural and organizational contexts, building on facilitating factors while being cognisant of those factors that may limit implementation.

While the case study focused only on implementing the competencies in two European countries, there are insights from their experience that can inform future implementation and research on progress in other countries. For example, the findings on the factors influencing implementation of the competencies at country level, in particular when combined with those of the online survey on individual decision-making, give an appreciation of some of the complexities that need to be addressed at professional and organizational levels in terms of leadership, norms, structures and processes in both future implementation and research on progress. The findings also provide some encouragement for the future in terms of how implementation can be advanced when the appropriate supports are put in place.

In terms of future research, the case study begins to address the gap in the literature on evaluating the take-up, use and impact of Health Promotion competencies but further, more in-depth exploration will be required as the uptake of the competencies expands to other countries and regions. Ongoing contemporary documentation of the implementation process in specific country contexts could provide valuable information concerning the effective adoption and integration of Health Promotion competencies across diverse organizational contexts and health systems. The methods used and the lessons learned in undertaking this case study offer a starting point for such future research in this under-explored aspect of Health Promotion.

### Strengths and Limitations of the Study

This study builds upon established theoretical frameworks in terms of case study methodology and implementation research and is innovative in providing an in-depth exploration of the use and impact of Health Promotion competencies at a country level. The study also forms part of a larger evaluation study that begins to address the gap in empirical studies on how Health Promotion competencies are used and what impact they have on Health Promotion practice, education and training.

The strengths of the study were enhanced by the openness and generosity of all informants, the breadth and quality of the information they provided and the time they dedicated to supporting the research.

The use of a case study approach allowed for exploration of the contextual factors influencing the implementation of the competencies with an emphasis on informants’ experience of using the competencies within different settings and system.

While additional resources might have allowed for more extensive interviews, it was considered that the data collected gives useful insight into the realities of implementing competencies in both countries. Given the relatively small number of Health Promotion specific practitioners and academics in both countries and the fact that as interviews progressed, little new data were forthcoming, the researchers were satisfied with the level of saturation achieved.

However, there are limitations in this study that should be noted. As with all qualitative research, the findings are not generalizable, and the data as analyzed by the researchers may have resulted in a particular interpretation of the implementation of the competencies and/or the status of Health Promotion in one or both countries. To counteract this, however, interpretation of the data was validated by the National Reference Group of experts in both countries to ensure accuracy.

As resources were limited, the interviews were conducted in English and this may have excluded some useful informants and may also have limited the flow of the interviews conducted in Italy, despite the excellent language skills of those interviewed. 

Finally, the researchers’ involvement in the development and operationalization of the competencies may have influenced how informants framed their responses and led to some bias in the responses received and/or in analysis of the data.

## 5. Conclusions

The findings of the case study show differences in the rate and focus of the implementation of the CompHP Competencies in Ireland and Italy that are influenced by a number of contextual factors reflecting the levels of Health Promotion infrastructure and capacity development in each country. A lack of awareness was identified as a major limiting factor in implementing the competencies in both countries, of particular concern in relation to employers and decision-makers, highlighting the need to disseminate information and advocate for their implementation.

The differences observed with regard to which contextual factors were found to influence the implementation of the competencies in the differing political, social, academic, practice and professional contexts in Ireland and Italy, provide important insights for their future implementation in other countries and for further research evaluating their uptake and impact.

## Figures and Tables

**Table 1:** Health Promotion infrastructure and capacity in Ireland and Italy.

	Ireland	Italy
Policy context	● Strategic framework: Healthy Ireland [35] Vision: A Healthy Ireland, where everyone can enjoy physical and mental health and wellbeing to their full potential. ● Minister for Health Promotion and the National Drugs Strategy	● Strategic framework: Guadagnare Salute—Rendere facili le scelte salutari [36] integrated into National and Regional Prevention Plans. Primary objective: to prevent and change unhealthy conducts that are the main risk factors for major non-communicable diseases
Implementation of Health Promotion	● Health and Wellbeing Division (HSE) [37] ● NGOs with remit for Health Promotion (e.g., [38,39,40]) the private sector	● Some evidence of implementation at national/regional level
Specialized Health Promotion workforce	● Specialized Health Promotion workforce circa 250 across the HSE, NGOs and private sector	● Very limited Health Promotion specific workforce/no specific career path
Health Promotion Academic Education	● Three courses accredited by the IUHPE (two undergraduate/one postgraduate) [41] ● Two other Health Promotion specific courses [42,43]	● Two courses accredited by the IUHPE (one undergraduate/one postgraduate [41] ● One course specific to Health Promotion) ● In 2019, the Ministry of University and Research included Health Promotion as a topic for post graduate courses for 22 non-medical health professions at national level [44]
National Professional Associations	● Association for Health Promotion Ireland (AHPI) [45]	● Health Promotion Group in Public Health Society [46] ● Italian Society for the Promotion of Health (SiPs) [47]
National Accreditation Organization (NAO) [48]	● NAO (Established 2016) ● Individual practitioners registered by Irish NAO	● Work ongoing on developing NAO ● Individual practitioners registered at IUHPE global level
Professional status	● No current statutory recognition of Health Promotion but interest in this for the future ● Voluntary registration (Irish NAO) ● Practitioners currently not required to be on any professional statutory register to be employed in Health Promotion	● No current statutory recognition of Health Promotion and unlikely to be in future ● Voluntary registration (IUHPE Global) ● Practitioners usually required to be on statutory register in relevant profession (e.g., medicine/psychology) to be employed in Health Promotion
Other dedicated Health Promotion organizations	Research ● Health Promotion Research Centre, NUIG [49]	Policy, research and evaluation ● National Center for Disease Prevention and Health Promotion [50] Research, education and training ● Experimental Center for Health Promotion and Health Education—CeSPES [51] Knowledge transfer and training ● Regional Documentation Center for Health Promotion—DoRs [52]

**Table 2:** Perceived impact of the CompHP Competencies in Ireland and Italy.

	Ireland	Italy
Health Promotion Education	Academic courses enhanced in terms of: ○ Development ○ Quality/ethos ○ Recruitment ○ Marketability Enhanced: ○ Students’ learning ○ Lecturers’ credibility ○ Graduates sense of Health Promotion role	Academic/training courses enhanced in terms of: ○ Development ○ Quality ○ Marketability Enhanced: ○ Students’ learning ○ Graduates’ knowledge/confidence
Health Promotion Profession	Profession ○ Strengthened recognition of profession NAO ○ Basis for NAO development and growth Professional Association ○ Helped consolidate, refocus organization ○ Helped ’sell’ Health Promotion to decision-makers/employers Workforce/practitioners ○ Engendered sense of professional community	NAO ○ Basis for developing NAO Workforce/practitioners ○ Supported recognition of Health Promotion roles (health professionals) ○ Basis for research resulting in changes in Health Promotion workforce.
Health Promotion Practice	Quality assurance ○ Used as structure/clear pathway for good practice ○ Facilitated holistic focus on Health Promotion ○ Provided clarity of role for workforce ○ Ensured all using the same language.	Quality assurance ○ Provided guide/checklist for good practice ○ Clarified Health Promotion roles ○ Supported reflection. ○ Informed planning at regional level.

**Table 3:** Advice on implementing the CompHP Competencies.

	Ireland	Italy
Key requirement	Knowledge and deep understanding of the competencies	Knowledge and understanding of Health Promotion
Practical advice	● Reflect on what is already being done and link the competencies to that - it’s less threatening ● Get buy-in from the wider workforce ● CompHP Handbooks/IUHPE website are key resources ● Gather peers and brainstorm what implementation will look like	● Link implementation to other relevant developments ● Ensure implementation is a bottom-up process ● Select examples of best practice in implementation and share with decision-makers ● Talk to those who have already implemented them ● Create enthusiasm
